# Filtering Empty Video Frames for Efficient Real-Time Object Detection

**DOI:** 10.3390/s24103025

**Published:** 2024-05-10

**Authors:** Yu Liu, Kyoung-Don Kang

**Affiliations:** Department of Computer Science, State University of New York at Binghamton, 4400 Vestal Parkway East, Vestal, NY 13850, USA; yliu456@binghamton.edu

**Keywords:** real-time object detection, filtering, frame processing rate, scalability, long short-term memory

## Abstract

Deep learning models have significantly improved object detection, which is essential for visual sensing. However, their increasing complexity results in higher latency and resource consumption, making real-time object detection challenging. In order to address the challenge, we propose a new lightweight filtering method called L-filter to predict empty video frames that include no object of interest (e.g., vehicles) with high accuracy via hybrid time series analysis. L-filter drops those frames deemed empty and conducts object detection for nonempty frames only, significantly enhancing the frame processing rate and scalability of real-time object detection. Our evaluation demonstrates that L-filter improves the frame processing rate by 31–47% for a single traffic video stream compared to three standalone state-of-the-art object detection models without L-filter. Additionally, L-filter significantly enhances scalability; it can process up to six concurrent video streams in one commodity GPU, supporting over 57 fps per stream, by working alongside the fastest object detection model among the three models.

## 1. Introduction

Real-time object detection is crucial for visual sensing using cameras in key IoT (Internet of Things) applications with great societal impact, such as smart transportation, surveillance, and manufacturing. State-of-the-art models based on deep learning, such as R-CNN (region-based convolutional neural network) [1], Fast R-CNN [2], Faster R-CNN [3], EfficientDet [4], SSD (single-shot multibox detector) [5], and YOLO models [6,7], effectively analyze visual sensor data. They have significantly enhanced the quality of object detection, such as accuracy, precision, and recall.

The increasing complexity of deep learning models, however, makes real-time object detection—which requires the minimum processing rate of 30 fps (frames per second)—challenging. This is a serious issue, since outdated sensor data analysis results may considerably degrade the effectiveness of decision-making in critical IoT applications, such as smart transportation and surveillance.

In order to address the issue, we previously introduced a lightweight CNN model called ERD (empty road detection) [8], which has considerably enhanced the frame processing rate of object detection in the context of real-time traffic monitoring. ERD is a CNN that preprocesses every video frame to detect if there is any object of interest, such as a vehicle, motorcycle, or pedestrian, in the frame. ERD filters empty frames with no object of interest and forwards only nonempty frames to the object detection model to improve the end-to-end frame processing rate.

ERD has a high classification accuracy of 0.96 [8] and significantly enhances the frame processing rate (fps) of object detection. Being a preprocessing method, ERD works alongside any object detection model. Thus, it is orthogonal and complementary to object detection. As ERD only performs binary classification (the road being empty or not), it is much smaller and faster compared to various lightweight CNN models, such as MobileNetV3 [9], ShuffleNetV2 [10], SqueezeNet [11], EfficientNetV2 [12], and InceptionV3 [13], as summarized in Table 1.

The CNN-based ERD model, however, has a limitation; when most frames are nonempty, for example, during rush hour, its overhead for analyzing every frame cannot be amortized by dropping many empty frames. The main contribution of this paper is the significant enhancement of the fps and scalability of real-time object detection by substantially extending ERD [8]. We introduce a new, lightweight hybrid time series analysis method called L-filter, which extends ERD by integrating it with a specially designed LSTM (long short-term memory) model. L-filter (L stands for LSTM) operates the ERD model periodically (e.g., once per second), recognizing that consecutive frames may exhibit similarity, and conducting dense pixel analysis for every frame (via ERD) is computationally intensive. For frames occurring between two periodic ERD executions, L-filter determines their occupancy status by analyzing concise numerical time series data instead of directly analyzing pixels. This approach substantially decreases overhead while maintaining higher accuracy compared to ERD, as it conducts time series analysis rather than analyzing dense pixels frame by frame without considering their similarity over short time intervals. Thereby, our approach boosts the frame processing rate and allows the system to conduct real-time object detection for multiple traffic video streams concurrently, improving the scalability crucial in real-world situations. For example, one real-time object detection system can simultaneously monitor several directions in a traffic intersection instead of requiring a dedicated machine in each direction.

Our evaluation reveals that the classification accuracy of L-filter for inferring the road occupancy status (empty or not) is 0.99, which is higher than that of RNN (recurrent neural network) [14], ERD [8], background subtraction [15], and ARIMA (autoregressive integrated moving average) [16] methods by 1–18%. Our results show that L-filter boosts the fps of three state-of-the-art (SOTA) object detection models: YOLOv5 [6], SSD [5], and EfficientDet [4] by 31–47%, respectively, while ERD improves them by 10–44%. Remarkably, L-filter combined with YOLOv5, the fastest model among the three object detection models, handles up to six concurrent streams, delivering over 57 fps per stream in a single consumer GPU (NVIDIA GeForce RTX 3080Ti). This signifies a total of 342 fps, which is 3.79× higher than that of ERD combined with YOLOv5, which achieves a total of 90 fps for a maximum of three concurrent streams.

The rest of the paper is organized as follows. In Section 2, related work is discussed. In Section 3, we briefly review ERD and introduce L-filter. The datasets used in this paper are described in Section 4. In Section 5, the performance of L-filter is compared to ERD and three orthogonal SOTA object detection models. Section 6 provides a summary of the key observations that verify the cost-effectiveness of L-filter, and we discuss our limitations and future research issues. Finally, we conclude the paper in Section 7.

## 2. Related Work

Object detection has been well studied with a long history of research. Recently, CNN-based object detection models have significantly outperformed traditional algorithms not based on deep learning, including [15,17,18,19]. (Due to the wealth of research on object detection, we only discuss works closely related to ours. Thus, our discussion in this section is neither complete nor comprehensive.)

Object detection using CNNs can be classified into two-stage and one-stage methods. Two-stage detectors, such as SPPNet [20], R-CNN [1], Fast R-CNN [2], Faster R-CNN [3], masked R-CNN [21], FPN [22], cascade R-CNN [23], and Libra R-CNN [24], use a region proposal network to generate the regions of interest (RoI) in the first stage. They perform bounding-box regression and object classification for the RoI in the second stage.

In contrast, single-stage detectors, such as SSD [5], EfficientDet [4], a series of YOLO models [6,7,25,26,27,28,29,30,31], Retina-net [32], CornerNet [33], CenterNet [34], and FCOS [35], predict bounding boxes and object classification in a single stage. Therefore, they are more effective for real-time applications. In general, newer models keep enhancing object detection accuracy at the cost of increasing complexity, as demonstrated, for example, in the YOLO models.

Novel vision transformers, such as [36,37,38,39], considerably improve the inference quality (e.g., accuracy) of vision tasks. Transformer-based object detection models include DETR [40], Deformable DETR [41], ViT-FRCNN [42], YOLOS [43], and ViTDet [44]. However, self-attention, which is the backbone of vision transformers, suffers from quadratic computational complexity and memory footprint.

In [45], a YOLO model has been pruned to make the model smaller. Lin et al. [46] applied advanced neural architecture search techniques to make computer vision tasks run on tiny IoT devices. However, model compression [47] usually decreases the inference accuracy. Thus, we do not adopt a compressed model in this paper.

Overall, we introduce a new filtering method that is complementary to object detection research, which is briefly reviewed in this section. In contrast to most of the existing work on object detection, we aim to (1) enhance the real-time frame processing rate by dropping empty frames that do not contribute to object detection and (2) support scalable real-time object detection for multiple concurrent video streams by utilizing otherwise idle resources in the GPU.

## 3. Empty Road Detection and L-Filter

In this section, we give a concise review of the ERD model [8]. Moreover, we introduce the L-filter framework to significantly boost the fps and scalability of real-time object detection while minimizing overhead.

### 3.1. ERD Model

**Figure 1:**
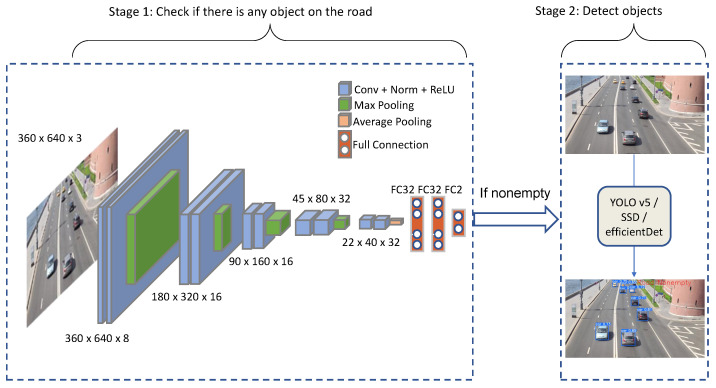
Object detection pipeline, consisting of ERD and an orthogonal object detection model.

Figure 1 depicts the object detection pipeline that integrates ERD [8] and an object detection model, such as EfficientDet, SSD, or YOLO. In a nutshell, the pipeline works as follows:

1. Execute the ERD model to infer whether the current traffic video frame is empty;
2. If the frame is nonempty, detect objects in the frame. Else, skip it;
3. Repeat the procedure for every frame.

In general, there is a tradeoff between prediction accuracy and latency. In order to strike a balance between the potentially conflicting requirements for high accuracy and the low latency of road occupancy classification, we have explored a comprehensive set of CNN architectures with different depths and widths to find the CNN architecture with as high accuracy and low latency as possible.

As a result, we have found the ERD model architecture in Figure 1. ERD consists of 13 layers that are grouped into six blocks: the first five blocks are convolutional, while the sixth block consists of the three fully-connected layers. Batch normalization and the rectified linear unit (ReLU) activation function are applied in each convolution layer to speed up convergence in training, mitigating possible gradient dispersion (Max/average pooling, being parameterless, is not considered a separate layer).

Being a binary classification model, ERD has 146,000 parameters only; it is smaller than the lightweight CNN models in Table 1 by one or two orders of magnitude. For more details on the ERD model, please refer to [8].

### 3.2. L-Filter

A disadvantage of ERD is the overhead needed to analyze dense pixels in every frame, as discussed before. In order to alleviate the issue, L-filter takes a hybrid approach:

- Our LSTM model conducts a time series analysis of concise numerical data in a sliding window representing the road occupancy status.
- It also triggers the ERD model every N>1 frames to decrease the frequency of ERD executions by *N* times, while effectively calibrating the predictions made by the LSTM model.

Algorithm 1 outlines how L-filter works. In the algorithm, *W* and *N* represent the sliding window size and the ERD execution period, respectively. In lines 1–3, for the first *W* frames, L-filter executes ERD and performs object detection using an object detection model, such as a YOLO model. During the $i^{th}$ iteration, as specified in lines 2–3, we store the binary classification result in B[i] and object count, i.e., the number of the detected objects, in C[i] for the $i^{th}$ frame. Thus, L-filter works the same way as ERD does for the first *W* frames and stores the numerical results in B[i] and C[i].

Beginning from the ${\left(W+1\right)}^{th}$ frame, L-filter works more efficiently than ERD does, as specified in lines 4–13, which is the main difference between ERD [8] and L-filter. In lines 5–6, L-filter uses ERD to classify every $N^{th}$ frame as empty or nonempty (0 or 1) and stores the result in B[i]. In lines 7–8, however, L-filter uses our LSTM model to analyze the other frames that are not the $kN^{th}$ frame, where k≥1. In line 8, to classify the $i^{th}$ frame, our LSTM model analyzes the previous binary classification results, B[i−W, …, i−1], and object counts, C[i−W, …, i−1], in the sliding window of size *W*. It stores the result of the classifying frame *i* in B[i].
**Algorithm 1:** L-filter to predict empty frames (based on the recent history in the sliding window)
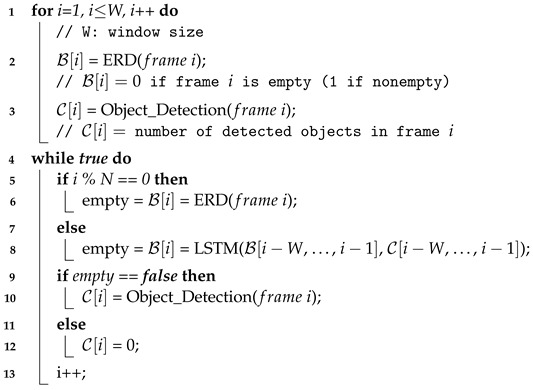


In lines 5–8, we enhance both the speed and accuracy of road occupancy classification instead of doing a tradeoff between them. In lines 5–6, we reduce the classification latency by periodically performing ERD. In lines 7–8, we aim to improve the accuracy by analyzing the sequence data that capture the potential similarity between the frames in the sliding window of size *W*. This is different from ERD, which analyzes each frame individually without considering the possible relationships between consecutive frames. Moreover, L-filter improves scalability by utilizing GPU resources (e.g., memory and stream multiprocessors), which are saved by skipping object detection for empty frames to analyze more traffic video streams concurrently.

In lines 9–10, if ERD or LSTM has inferred that frame *i* is nonempty, L-filter conducts object detection. The object detection model displays bounding boxes and provides the number of detected objects. In line 10, L-filter stores the object count in C[i]. On the other hand, if the frame is empty, L-filter stores 0 in C[i] in line 12. Finally, the frame number is incremented by 1 in line 13, and lines 5–13 are repeated for the next video frames. If L-filter concurrently processes multiple traffic video streams, it applies Algorithm 1 to each stream.

### 3.3. LTSM Model for Road Occupancy Predictions

In this paper, we devise a specialized LSTM model to efficiently infer road occupancy status with high accuracy. We use LSTM, as it is known to be very effective for sequence data predictions [48]. It mitigates the limitations of RNNs [14], such as short-term information maintenance and vanishing gradients, through the use of memory cells.

**Figure 2:**
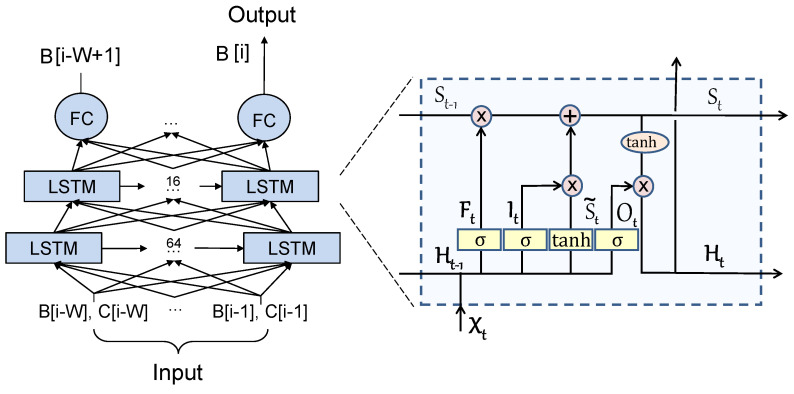
Architecture of the LSTM in L-filter for classifying frame *i* as empty or not.

In particular, our objective is to support higher accuracy as well as shorter latency compared to ERD. To this end, we have explored various LSTM architectures to maximize accuracy while decreasing the complexity of the network structure as much as possible. In this way, we have designed the LSTM model, as depicted in Figure 2. Given the input sequence data in the sliding window of size *W*, our LSTM model in Figure 2 infers the road occupancy status of frame *i* and outputs the inference result, i.e., B[i], without doing dense pixel analysis, which is different from ERD. The model consists of two LSTM layers and one fully connected (FC) layer that uses a sigmoid function for activation. The first LSTM layer has a hidden state vector of size 64, while the second layer has a hidden state vector of size 16.

Within the recurrent hidden layers, LSTM incorporates specialized memory blocks. In each LSTM cell, as illustrated on the right side in Figure 2, these blocks house memory cells with self-connections, allowing them to preserve the temporal state of the network at any moment, *t*, between frame i−W and frame i−1. Additionally, every memory block contains unique multiplicative units known as gates: input gate, output gate, and forget gate. In Figure 2 and Equations (1)–(6), $X_{t}$, $H_{t}$, and $S_{t}$ represent the input, hidden state, and cell state, respectively, of the LSTM cell at time step *t*. The input gate ($I_{t}$) regulates the flow of input activations into the memory cell. Similarly, the output gate ($O_{t}$) governs the flow of output into the rest of the network. Meanwhile, the forget gate ($F_{t}$) adjusts the internal state of the memory cell before incorporating it, thus enabling adaptive memory retention. $W_{f}$, $W_{i}$, $W_{s}$, $W_{o}$, $b_{f}$, $b_{i}$, $b_{s}$, and $b_{o}$ represent the learnable parameters and bias terms associated with the forget gate, input gate, candidate cell state, and output gate, respectively. σ is the sigmoid function, tanh is the hyperbolic tangent, and ⊙ is the Hadamard (elementwise) product.

(1) $$\mathrm{Forget}\;\mathrm{Gate}:\;F_{t}=\sigma \left(W_{f}\cdot \left[H_{t-1},X_{t}\right]+b_{f}\right)$$

(2) $$\mathrm{Input}\;\mathrm{Gate}:\;I_{t}=\sigma \left(W_{i}\cdot \left[H_{t-1},X_{t}\right]+b_{i}\right)$$

(3) $$\mathrm{Cell}\;\mathrm{State}:\;\tilde{S}=\tanh\left(W_{s}\cdot \left[H_{t-1},X_{t}\right]+b_{s}\right)$$

(4) $$\mathrm{Updated}\;\mathrm{Cell}\;\mathrm{State}:\;S_{t}=F_{t}⊙S_{t-1}+I_{t}⊙{\tilde{S}}_{t}$$

(5) $$\mathrm{Output}\;\mathrm{Gate}:\;O_{t}=\sigma \left(W_{o}\cdot \left[H_{t-1},X_{t}\right]+b_{o}\right)$$

(6) $$\mathrm{Hidden}\;\mathrm{State}:\;H_{t}=O_{t}⊙\tanh\left(S_{t}\right)$$

By effectively analyzing the sequence data in the sliding window, our LSTM model boosts accuracy compared to ERD while reducing latency for road occupancy classification. Furthermore, we have considered the background subtraction [15], ARIMA [16], and RNN [14] methods to infer road occupancy. However, we have selected LSTM due to its superior accuracy. A more detailed discussion is given in Section 5.

## 4. Datasets, Model Training, and Implementation

In this section, we discuss the datasets used in this paper, the training of L-filter, and model implementation.

### 4.1. Datasets

Finding an appropriate dataset to evaluate ERD and L-filter is challenging:

- Many computer vision datasets, including [49,50,51], are used for specific tasks, such as object detection or segmentation. We have found that they have few empty frames, even though traffic videos may involve a large fraction of empty frames, especially during the off-peak time.
- Traffic datasets, including [52,53,54], do not label each frame as empty or not. Thus, computer vision or traffic datasets need to be labeled frame by frame, which is very time-consuming and costly.

In order to tackle these challenges, in this paper, we use two datasets called Dataset 1 and Dataset 2 hereafter.

#### 4.1.1. Dataset 1

We have generated Dataset 1 by manually labeling every frame in the DZ Computer Vision dataset [55] as empty or nonempty. The dataset contains images extracted from high-definition video with 1280×720 resolution captured on the road from the Bolshoy Moskvoretsky Bridge to the Kremlin Embankment in Moscow, Russia.

Through manual labeling, we have found that only 21% of the images in the dataset are empty. Thus, the labels are considerably imbalanced, which may lead to overfitting. In order to significantly reduce the risk of overfitting and enhance generalizability, we take several approaches:

- Data augmentation: We augment the dataset by synthesizing every image labeled 0 (empty) by rotating, flipping, changing lighting, and blurring them. As a result, the fraction of empty images with no object of interest has increased to 35%, obtaining a total of 6,438 images with no object of interest.
- Early stopping: We analyze training and validation losses over epochs and terminate training early as soon as the losses converge to small values and stabilize. (A more detailed discussion is given in Section 4.2).
- LSTM architecture search: In an iterative feedback loop, we vary the depth and width of the LSTM model of L-filter and analyze losses for different LSTM architectures. Thereby, we find a low-complexity LSTM model that converges to minimal validation as well as training losses in a small number of epochs.
- Generalizability analysis using a different dataset: In this paper, we train the LSTM of L-filter using Dataset 1; however, we analyze its possible impact on object detection performance using a different dataset—Dataset 2—in terms of mAP (mean average precision), which is a common metric for evaluating object detection.

#### 4.1.2. Dataset 2

In order to evaluate the object detection performance provided by L-filter, we used a public dataset [56]. Dataset 2 is annotated with ground truth bounding boxes that identify vehicles. Moreover, all the frames in this dataset are nonempty. The objectives of using this dataset follow:

- The first objective is to evaluate the generalizability of L-filter using Dataset 2, as discussed in Section 4.1.1. As Dataset 2 is already annotated with bounding boxes, we evaluate the mAP by comparing them to the bounding boxes provided by L-filter when it works alongside an object detection model, e.g., a YOLO model.
- Another objective is to analyze the worst-case overhead of L-filter using a real-world traffic video with no empty frame [56], which represents a heavy traffic scenario, where L-filter is subject to the highest overhead (the evaluation results are described in Section 5).

Dataset 2 has 499 annotated frames in total. All of them are used as a test set, because Dataset 2 is not used for training.

### 4.2. LSTM Training Using Dataset 1

Dataset 1 was used to train our LSTM model, and it has 20,595 frames in total. The frames are split into training, validation, and test sets, as specified in Table 2. (In this study, we used the pretrained ERD model [8]. For more details of ERD, interested readers are referred to [8]).

For L-filter, we captured the numeric features from every frame because LSTM needs sequential features. In particular, we created two feature vectors. As described in Section 3.2, the first vector represents road occupancy status, 0 or 1, for each frame in the dataset. The second feature vector records the object count in each frame obtained using an object detection model, e.g., YOLOv5. We divided both vectors into windows of size *W* each. We then combined road occupancy status and object count vectors into one matrix to form samples. By doing this, we generated a total of 20,595 samples, as is shown in Table 2.

In order to train the LSTM model of L-filter, we defined the MSE (mean squared error) loss function:

(7) $$L=\frac{1}{n}\sum_{i=1}^{n}{\left(y_{i}-B\left[i\right]\right)}^{2}$$

where $y_{i}$ is the ground truth that indicates if frame *i* is 0 or 1, B[i] is L-filter’s classification of frame *i*, and *n* is the number of samples in the training dataset. Hence, 0≤L≤1.

In order to minimize the loss, we used the Adam optimizer. Specifically, the optimizer tunes LSTM parameters by using the following equation:

(8) $$\theta_{i+1}=\theta_{i}-\frac{\eta }{\sqrt{{\hat{v}}_{i}}+\epsilon }{\hat{m}}_{i}$$

where η denotes the learning rate, $\hat{m_{i}}$ estimates the first momentum (mean) of gradients, $\hat{v_{i}}$ estimates the second momentum of gradients, and ϵ is a small constant added to prevent division by zero. In this paper, we set the learning rate η=0.001 and updated parameters for 5 epochs with a batch size of 8. Since there are 16,476 sample frames in the training and validation sets, as shown in Table 2, there are 2060 iterations per epoch. The 4119 samples in the test set were reserved for evaluation in Section 5.

Furthermore, we empirically configure the window size (*W*) and ERD execution period (*N*) in Algorithm 1. Specifically, we set W=5, which is the smallest window size that supports high inference accuracy. Similarly, we set N=30 frames to allow infrequent ERD executions while maintaining high accuracy.

**Figure 3:**
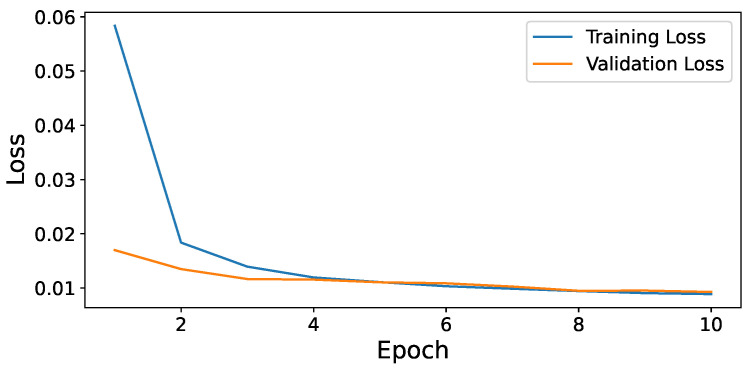
The training and validation losses of L-filter (Equation (7)) for training epochs.

By following the described training procedure, we gained insights into the training process by analyzing the MSE loss in Equation (7) for successive epochs. As depicted in Figure 3, we observe that the validation as well as training loss curves keep descending until they reach a plateau after the 5*^th^* epoch. As the figure shows a desirable pattern of consistently decreasing losses that quickly stabilize, we halted the training early at epoch 5 to prevent overfitting. Furthermore, to minimize the risks of both underfitting and overfitting, we fine-tuned the L-filter complexity by adjusting the number of layers and neurons while analyzing their losses over epochs, as discussed above. By repeating this process, we derived the architecture of the L-filter, as illustrated in Figure 2.

### 4.3. Implementation

Our preprocessing system is flexible in that it can work with any object detection model to enhance efficiency by reliably removing empty frames. For the evaluation, we used EfficientDet [4], SSD [5], and YOLOv5 [25], which are effective single-stage object detection models. We selected them for their higher inference speed when compared to similar detection accuracy in two-stage object detection models, such as R-CNN [1], Fast R-CNN [2], and Faster R-CNN [3]. Specifically, we chose EfficientDet-B0, SSD300, and YOLOv5s for our study because they are smaller than their variants while still achieving high-quality object detection.

For our evaluation, we used a workstation with an Intel^®^ Core i7-7820X CPU, 64 GB RAM, and an NVIDIA GeForce RTX 3080Ti GPU. It mimics an edge server that supports object detection at the edge rather than sending all visual sensor data (video frames) to the cloud, incurring high latency and potential congestion in the Internet core. The operating system is Ubuntu 18.04.6 LTS. We have used Python 3.9, PyTorch 1.13, and OpenCV-python 4.6.0 [57] to implement and evaluate the deep learning models.

## 5. Evaluation

In this section, the frame processing rate (fps), scalability, and mean average precision (mAP) are evaluated by using the test sets that were unseen by the models during model training. Specifically, the test set of Dataset 1 was used in Section 5.1, Section 5.2 and Section 5.3, while both the test set of Dataset 1 and the entirety of Dataset 2 were used for evaluation in Section 5.4.

### 5.1. Road Occupancy Classification Accuracy

**Figure 4:**
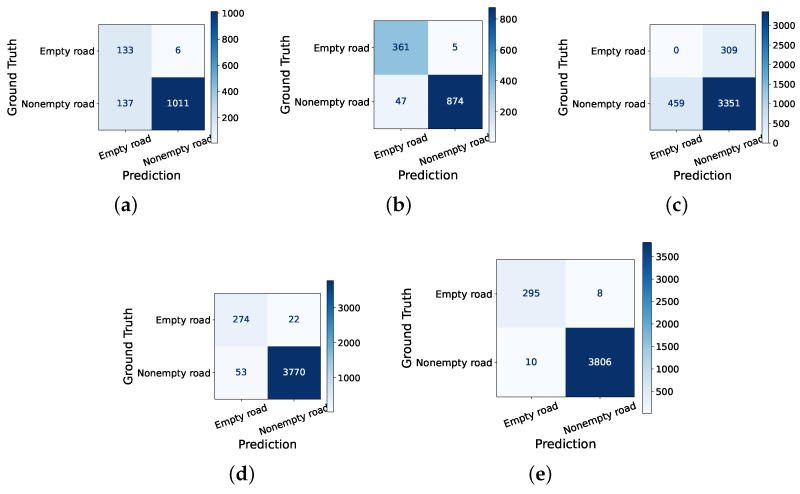
Confusion matrix of road occupancy predictions. (**a**) Background subtraction: Accuracy: 0.89, recall: 0.88, precision: 0.99, and F1-score: 0.93. (**b**) ARIMA. Accuracy: 0.81, recall: 0.87, precision: 0.91, and F1-score: 0.89. (**c**) RNN. Accuracy: 0.98, recall: 0.98, precision: 0.99, and F1-score: 0.99. (**d**) ERD. Accuracy: 0.96, recall: 0.95, precision: 0.97, and F1-score: 0.97. (**e**) L-filter. Accuracy: 0.99, recall: 0.99, precision: 0.99, and F1-score: 0.99.

Figure 4 illustrates the road occupancy classification performance of background subtraction [15], ARIMA [16], RNN [14], ERD, and L-filter. As shown in the figure, our LSTM model of L-filter achieves the highest accuracy, precision, recall, and F1-score, as it conducts hybrid sequence analysis that combines periodic ERD executions and LSTM-based predictions in between. The inference performance of the other methods in Figure 4 is lower than that of L-filter for the following potential reasons. Background subtraction is not robust to noise, such as trees in the wind [58]. ARIMA suffers from poor performance since it assumes a stationary time series, where statistical properties, such as the mean and variance, are assumed to remain constant over time [59]. However, the assumption may not hold in dynamic traffic scenarios. Although comparable to L-filter, the inference performance of RNN is slightly lower. In general, RNN is not as effective as LSTM in grasping prolonged dependencies in sequential data and is subject to vanishing gradient problems [48]. Thus, we utilized LSTM instead of RNN in this paper.

Furthermore, as shown in Table 3, the latency of L-filter is only 45% of that of ERD. It is only about 12%, 5%, and 2% of the latency for object detection in YOLOv5, SSD300, and EfficientDet-B0, respectively. Thus, L-filter is cost-effective.

### 5.2. Frame Processing Rate Enhancements by Erd and L-Filter for One Traffic Video Stream

As shown in Table 4, when ERD runs alongside an object detection model, it enhances the fps of YOLOv5, SSD, and EfficientDet by approximately 10–44%. In the table, L-filter outperforms ERD by improving the fps of the three tested object detection models by roughly 31–47%. L-filter outperforms ERD more noticeably when it is paired with YOLOv5; that is, it is the fastest among the three tested object detection models. This is because the lower overhead of L-filter (than that of ERD) becomes most impactful in terms of fps improvement when it is integrated with the fastest object detection model (A more detailed overhead analysis of ERD and L-filter is given in Section 5.4).

### 5.3. Scalability of ERD and L-Filter for Concurrent Streams

In this subsection, we evaluate how many concurrent traffic video streams ERD+YOLOv5 and L-filter+YOLOv5 can process while supporting at least 30 fps per stream, which is required for real-time object detection.

As summarized in Table 5, ERD can concurrently process up to three streams with at least 30 fps per stream before depleting the GPU memory. Thus, its aggregate frame processing rate for three streams is over 91 fps.

Markedly, L-filter+YOLOv5 processes up to six concurrent streams, supporting over 57 fps per stream, as illustrated in Table 6. The aggregate fps achieved by L-filter+YOLO for six streams exceeds 345 fps, which is more than 3.79× the total fps supported by ERD+YOLOv5 for three streams. Thus, L-filter significantly upgrades the scalability of real-time object detection.

### 5.4. mAP and Overhead of L-Filter

For the clarity of the presentation, we use a single video stream to analyze the mAP and overhead of L-filter in this subsection.

#### 5.4.1. mAP

By using Dataset 2 (Section 4.1.2), which was not utilized for training the LSTM model of L-filter, we analyze the mAP and generalizability of L-filter. As shown in Table 7, the mAP of L-filter+YOLOv5 is comparable to that of YOLOv5 for different IoU (Intersection over Union) thresholds. These results reveal that L-filter supports acceptable object detection performance when compared to YOLOv5. Moreover, it is generalizable to different datasets of traffic surveillance.

#### 5.4.2. Overhead

In order to estimate the worst-case overhead of L-filter in an extreme case with all nonempty frames, we assess the frame processing rate using Dataset 2. The YOLOv5 without L-filter supports 56.5 fps, whereas YOLOv5+L-filter provides 51.2 fps. Thus, the observed overhead of L-filter is 9.38% for Dataset 2.

**Figure 5:**
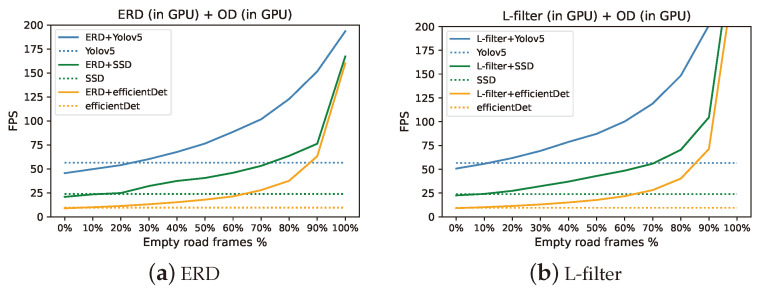
Percentage of empty frames vs. fps.

Additionally, we adjusted the proportion of empty frames in Dataset 1 via data augmentation (Section 4.1.1) to undertake a further cost-benefit analysis of ERD and L-filter. Specifically, we evaluated the fps for different proportions of empty frames in Dataset 1, ranging from 0% to 100%, with a 10% increment. In Figure 5, each dotted horizontal line shows the fps of an independent object detection model without ERD or L-filter. They are not affected by the proportion of empty frames in the traffic video. Thus, their fps values in Figure 5 are constant, regardless of the fraction of empty frames in the traffic monitoring video. As depicted in Figure 5, ERD or L-filter conjoined with an object detection model achieves a higher fps than the corresponding object detection model does on its own when the percentage of empty frames is beyond a break-even threshold or vice versa. Thus, the lower the threshold, the better.

In Table 8, the break-even threshold of L-filter is lower than that of ERD by approximately 47–52%. Therefore, L-filter is significantly more efficient than ERD. In addition, we observe that the slowest EfficientDet model and the fastest YOLOv5 model have the lowest and highest threshold, respectively. This is because the ratio of ERD or L-filter execution time to that of a faster model is greater.

As shown in Figure 5 and Table 9, the fps monotonically increases as the proportion of empty frames in the video increases. When there is no empty frame in the traffic video stream, the 50.7 fps provided by L-filter+YOLOv5 in Table 9 is similar to the 51.2 fps previously observed for Dataset 2, which lacks empty frames. For the entire range of empty frames, L-filter achieves higher fps than ERD does. Furthermore, the enhancement upon ERD grows as the fraction of empty frames increases.

## 6. Discussion

Enhancing the efficiency and scalability of real-time object detection is still an open problem. SOTA deep learning models for object detection, including the models discussed in Section 2, improve object detection performance at the cost of increasing model complexities and resource requirements. In order to shed light on this issue, as described in Section 3, L-filter predicts empty video frames that include no object of interest, such as a vehicle, via a hybrid time series analysis. It skips frames deemed empty to boost the efficiency and scalability of real-time object detection. In our LSTM model training, we systematically applied SOTA approaches to minimize the risk of overfitting while striving to enhance generalizability. In addition, we verified that the training and validation losses converge reliably, as discussed in Section 4.

Our evaluation results in Section 5 verify the cost-effectiveness of the L-filter design:

- L-filter increases the accuracy of road occupancy classification by 1–18% compared to ERD and the other tested classification baselines;
- The object detection performance of L-filter+YOLOv5, in terms of mAP, is comparable to that of YOLOv5;
- L-filter enhances the fps of real-time object detection for a single video stream by 31–47% compared to the three effective object detection models;
- Without depleting GPU resources, L-filter can process up to six concurrent video streams in a consumer GPU, supporting more than 57 fps per stream. Thus, it can significantly reduce the cost for deploying AI-based traffic surveillance systems in a smart city.

L-filter, however, incurs overhead when the traffic surveillance video has fewer empty frames. Related future research directions toward further reducing the overhead include the following:

- The diurnal and seasonal traffic patterns can be leveraged to dynamically turn on L-filter only when it is expected to be effective; that is, the expected number of empty frames is higher than the break-even threshold. Accurate predictions, however, are challenging in the presence of unpredictable traffic incidents. Care should be taken to immediately turn L-filter off when an incident that can cause an abnormal traffic pattern occurs.
- Another approach to reduce the overhead could be integrating L-filter with an object detection model, e.g., a YOLO model, and make them share certain layers instead of utilizing L-filter as a separate unit for preprocessing. An effective design and fine-tuning of an integrated model represent the main challenge in this approach.

Another challenge is the lack of datasets, wherein each frame is labeled as empty or nonempty. A possible approach to alleviating this issue in the future is leveraging emerging technology for synthetically generating realistic data [60] instead of manually labeling video frames.

In this paper, we focus on enhancing the efficiency and scalability of generic object detection. Small object detection [61,62] is important for remote (e.g., aerial/maritime) sensing and medical image analysis, but it has not been considered. Improving both the accuracy and efficiency of small object detection remains an open problem that requires more in-depth research. Furthermore, in this paper, we focus on efficiently detecting predefined objects of interest, such as vehicles, in real time. Objects that are not predefined, e.g., a small bird, are out of interest. A potential extension in the future would allow a user to reconfigure objects of interest according to the application at hand. For example, a user can specify birds, drones, and other features to identify drones in a restricted flight zone [61,63]. An end-to-end framework that enables the configuration, model design, training/fine-tuning, and efficient real-time inference will be desirable.

A thorough investigation of these research issues is beyond the scope of this paper and is reserved for future work.

## 7. Conclusions

Recent advancements in deep learning have significantly improved object detection. Those models, however, have grown in complexity, leading to challenges in terms of supporting the required frame processing rate of at least 30 fps and enhancing the scalability of real-time object detection. In order to address the challenge, we propose a new lightweight filtering method called L-filter. Based on hybrid time series analysis, L-filter predicts empty video frames that do not include any object of interest (e.g., vehicles) with high accuracy. Subsequently, L-filter drops empty frames and performs object detection for nonempty frames only. Our evaluation validates the efficiency and scalability of L-filter, verifying its object detection performance when it works alongside an object detection model as a preprocessing unit for filtering empty frames. It demonstrates that L-filter improves the frame processing rate (fps) by 31–47% for a single traffic video stream when compared to three standalone object detection models without L-filter. L-filter, conjoined with YOLOv5, supports similar mean average precision to that of YOLOv5 without L-filter. Additionally, L-filter substantially improves the scalability of real-time object detection: it orchestrates object detection for up to six concurrent streams, supporting over 57 fps for each stream in a single commodity GPU. Encouraged by these promising results, we will continue exploring more advanced approaches to efficient real-time object detection, including the future work issues outlined in Section 6.

## Figures and Tables

**Table 1:** Comparisons of lightweight CNN models (GPU: NVIDIA RTX 3080Ti, CPU: Intel^®^ Core i7-7820X CPU). In this paper, a **boldface** number in a table indicates the most efficient one.

Models	Parameters	GFLOPs	Latency@GPU	Latency@CPU
MobileNetV3-Small	2.5 M	**0.27**	8.4 ms	14.2 ms
ShuffleNetV2-X0	2.3 M	0.71	9.3 ms	20.3 ms
SqueezeNet1.1	1.2 M	1.74	5.5 ms	22.5 ms
EfficientNet-B0	5.3 M	1.88	12.2 ms	46.1 ms
EfficientNetV2-Small	21.5 M	13.4	25.1 ms	94.5 ms
InceptionV3	23.8 M	15.01	18.6 ms	80.9 ms
ERD (our previous work)	**0.15 M**	0.54	**4.7 ms**	**10.2 ms**

**Table 2:** The number of frames in the training, validation, and test sets of Dataset 1.

Dataset	Training	Validation	Testing
Number	13,180	3296	4119

**Table 3:** Latency of L-filter, ERD, and the object detection models.

Model	Average Latency (ms)
L-filter	2.2
ERD	4.9
YOLOV5s	17.7
SSD300	42.0
EfficientDet-B0	105.3

**Table 4:** Frame processing rate improvements achieved by ERD and L-filter when they work alongside YOLOv5s, SSD300, and EffcientDet-B0, respectively. In each cell, the first number is the total frame processing rate (fps), and the second number in the parenthesis is the enhancement of the fps achieved by ERD and L-filter, respectively.

	YOLOv5	SSD	EfficientDet
ERD	62.4 (10.4%)	33.3 (39.9%)	13.7 (44.2%)
L-filter	**73.8 (30.6%)**	**33.6 (41.2%)**	**14 (47.4%)**

**Table 5:** Per-stream frame processing rate (fps) of ERD+YOLOv5 when one or more streams are processed concurrently.

Number of Streams	1	2	3
ERD+YOLOv5	62.4	53.1	30.6

**Table 6:** Per-stream frame processing rate (fps) of L-filter+YOLOv5 when one or more streams are processed concurrently.

Number of Streams	1	2	3	4	5	6
L-filter+YOLOv5	**73.8**	**70.1**	**65.5**	**63.4**	**60.2**	**57.6**

**Table 7:** mAP of object detection for different IoU thresholds.

IoU threshold	0.1	0.2	0.5
YOLOv5	0.442	0.441	**0.400**
L-filter+YOLOv5	**0.448**	**0.442**	**0.400**

**Table 8:** Break-even thresholds of ERD and L-filter.

	YOLOv5	SSD	EfficientDet
ERD	24.4%	17.3%	7.0%
L-filter	**11.4%**	**8.3%**	**3.7%**

**Table 9:** Proportion of empty frames vs. fps. In each cell, the first and second number are the fps when 0% and 100% of the frames are empty, respectively.

	YOLOv5	SSD	EfficientDet
ERD	45.5–193.5	20.8–167.2	8.9–160.0
L-filter	**50.7–374.6**	**22.5–311.5**	**9.1–271.7**

## Data Availability

Our source code and labeled dataset are available at: https://github.com/Real-Time-Lab/Filtering-Empty-Video-Frames-for-Efficient-Real-Time-Object-Detection (accessed on 1 April 2024).

## References

[1] Girshick R., Donahue J., Darrell T., Malik J. Rich feature hierarchies for accurate object detection and semantic segmentation. Proceedings of the IEEE Conference on Computer Vision and Pattern Recognition.

[2] Girshick R. Fast r-cnn. Proceedings of the IEEE International Conference on Computer Vision.

[3] Ren S., He K., Girshick R., Sun J. Faster r-cnn: Towards real-time object detection with region proposal networks. Proceedings of the Advances in Neural Information Processing Systems 28 (NIPS 2015).

[4] Tan M., Pang R., Le Q.V. Efficientdet: Scalable and efficient object detection. Proceedings of the IEEE/CVF Conference on Computer Vision and Pattern Recognition.

[5] Liu W., Anguelov D., Erhan D., Szegedy C., Reed S., Fu C.Y., Berg A.C. (2016). SSD: Single shot multibox detector. Proceedings of the Computer Vision–ECCV 2016: 14th European Conference.

[6] YOLOv5. https://github.com/ultralytics/yolov5/wiki.

[7] Bochkovskiy A., Wang C.Y., Liao H.Y.M. (2020). Yolov4: Optimal speed and accuracy of object detection. arXiv.

[8] Liu Y., Kang K.D. Preprocessing via Deep Learning for Enhancing Real-Time Performance of Object Detection. Proceedings of the IEEE 97th Vehicular Technology Conference (VTC2023-Spring).

[9] Howard A., Sandler M., Chu G., Chen L.C., Chen B., Tan M., Wang W., Zhu Y., Pang R., Vasudevan V. Searching for mobilenetv3. Proceedings of the IEEE/CVF International Conference on Computer Vision.

[10] Zhang X., Zhou X., Lin M., Sun J. Shufflenet: An extremely efficient convolutional neural network for mobile devices. Proceedings of the IEEE Conference on Computer Vision and Pattern Recognition.

[11] Iandola F.N., Han S., Moskewicz M.W., Ashraf K., Dally W.J., Keutzer K. (2016). SqueezeNet: AlexNet-level accuracy with 50x fewer parameters and <0.5 MB model size. arXiv.

[12] Tan M., Le Q. Efficientnet: Rethinking model scaling for convolutional neural networks. Proceedings of the International Conference on Machine Learning.

[13] Szegedy C., Vanhoucke V., Ioffe S., Shlens J., Wojna Z. Rethinking the inception architecture for computer vision. Proceedings of the IEEE Conference on Computer Vision and Pattern Recognition.

[14] Medsker L.R., Jain L. (2001). Recurrent Neural Networks: Design and Applications.

[15] Sen-Ching S.C., Kamath C. (2004). Robust techniques for background subtraction in urban traffic video. Proceedings of the Visual Communications and Image Processing.

[16] Box G.E., Jenkins G.M., Reinsel G.C., Ljung G.M. (2015). Time Series Analysis: Forecasting and Control.

[17] Lowe D.G. (2004). Distinctive image features from scale-invariant keypoints. Int. J. Comput. Vis..

[18] Zivkovic Z. Improved adaptive Gaussian mixture model for background subtraction. Proceedings of the 17th IEEE International Conference on Pattern Recognition.

[19] Dutt Jain S., Xiong B., Grauman K. Fusionseg: Learning to combine motion and appearance for fully automatic segmentation of generic objects in videos. Proceedings of the IEEE Conference on Computer Vision and Pattern Recognition.

[20] He K., Zhang X., Ren S., Sun J. (2015). Spatial pyramid pooling in deep convolutional networks for visual recognition. IEEE Trans. Pattern Anal. Mach. Intell..

[21] He K., Gkioxari G., Dollár P., Girshick R. Mask r-cnn. Proceedings of the IEEE International Conference on Computer Vision.

[22] Lin T.Y., Dollár P., Girshick R., He K., Hariharan B., Belongie S. Feature pyramid networks for object detection. Proceedings of the IEEE Conference on Computer Vision and Pattern Recognition.

[23] Cai Z., Vasconcelos N. (2019). Cascade R-CNN: High quality object detection and instance segmentation. IEEE Trans. Pattern Anal. Mach. Intell..

[24] Pang J., Chen K., Shi J., Feng H., Ouyang W., Lin D. Libra r-cnn: Towards balanced learning for object detection. Proceedings of the IEEE/CVF Conference on Computer Vision and Pattern Recognition.

[25] Redmon J., Farhadi A. YOLO9000: Better, faster, stronger. Proceedings of the IEEE Conference on Computer Vision and Pattern Recognition.

[26] Redmon J., Divvala S., Girshick R., Farhadi A. You only look once: Unified, real-time object detection. Proceedings of the IEEE Conference on Computer Vision and Pattern Recognition.

[27] Redmon J., Farhadi A. (2018). Yolov3: An incremental improvement. arXiv.

[28] Li C., Li L., Jiang H., Weng K., Geng Y., Li L., Ke Z., Li Q., Cheng M., Nie W. (2022). YOLOv6: A Single-Stage Object Detection Framework for Industrial Applications. arXiv.

[29] Wang C.Y., Bochkovskiy A., Liao H.Y.M. (2022). YOLOv7: Trainable bag-of-freebies sets new state-of-the-art for real-time object detectors. arXiv.

[30] Reis D., Kupec J., Hong J., Daoudi A. (2023). Real-Time Flying Object Detection with YOLOv8. arXiv.

[31] Wang C.Y., Yeh I.H., Liao H.Y.M. (2024). YOLOv9: Learning What You Want to Learn Using Programmable Gradient Information. arXiv.

[32] Lin T.Y., Goyal P., Girshick R., He K., Dollár P. Focal loss for dense object detection. Proceedings of the IEEE International Conference on Computer Vision.

[33] Law H., Deng J. Cornernet: Detecting objects as paired keypoints. Proceedings of the European Conference on Computer Vision (ECCV).

[34] Duan K., Bai S., Xie L., Qi H., Huang Q., Tian Q. Centernet: Keypoint triplets for object detection. Proceedings of the IEEE/CVF International Conference on Computer Vision.

[35] Tian Z., Shen C., Chen H., He T. (2020). FCOS: A simple and strong anchor-free object detector. IEEE Trans. Pattern Anal. Mach. Intell..

[36] Yin H., Vahdat A., Alvarez J.M., Mallya A., Kautz J., Molchanov P. AdaViT: Adaptive tokens for efficient vision transformer. Proceedings of the IEEE/CVF Conference on Computer Vision and Pattern Recognition.

[37] Dosovitskiy A., Beyer L., Kolesnikov A., Weissenborn D., Zhai X., Unterthiner T., Dehghani M., Minderer M., Heigold G., Gelly S. (2020). An image is worth 16×16 words: Transformers for image recognition at scale. arXiv.

[38] Touvron H., Cord M., Douze M., Massa F., Sablayrolles A., Jégou H. Training data-efficient image transformers & distillation through attention. Proceedings of the International Conference on Machine Learning.

[39] Liu Z., Lin Y., Cao Y., Hu H., Wei Y., Zhang Z., Lin S., Guo B. Swin transformer: Hierarchical vision transformer using shifted windows. Proceedings of the IEEE/CVF International Conference on Computer Vision.

[40] Carion N., Massa F., Synnaeve G., Usunier N., Kirillov A., Zagoruyko S. (2020). End-to-end object detection with transformers. Proceedings of the European Conference on Computer Vision.

[41] Zhu X., Su W., Lu L., Li B., Wang X., Dai J. (2020). Deformable detr: Deformable transformers for end-to-end object detection. arXiv.

[42] Beal J., Kim E., Tzeng E., Park D.H., Zhai A., Kislyuk D. (2020). Toward transformer-based object detection. arXiv.

[43] Fang Y., Liao B., Wang X., Fang J., Qi J., Wu R., Niu J., Liu W. You only look at one sequence: Rethinking transformer in vision through object detection. Proceedings of the Advances in Neural Information Processing Systems 34 (NIPS 2021).

[44] Li Y., Mao H., Girshick R., He K. (2022). Exploring plain vision transformer backbones for object detection. Proceedings of the European Conference on Computer Vision.

[45] Taheri Tajar A., Ramazani A., Mansoorizadeh M. (2021). A lightweight Tiny-YOLOv3 vehicle detection approach. J. Real-Time Image Process..

[46] Lin J., Chen W.M., Lin Y., Gan C., Han S. MCUNet: Tiny deep learning on IoT devices. Proceedings of the Advances in Neural Information Processing Systems 33 (NIPS 2020).

[47] Deng L., Li G., Han S., Shi L., Xie Y. (2020). Model Compression and Hardware Acceleration for Neural Networks: A Comprehensive Survey. Proc. IEEE.

[48] Yu Y., Si X., Hu C., Zhang J. (2019). A review of recurrent neural networks: LSTM cells and network architectures. Neural Comput..

[49] Geiger A., Lenz P., Stiller C., Urtasun R. (2013). Vision meets robotics: The kitti dataset. Int. J. Robot. Res..

[50] Wen L., Du D., Cai Z., Lei Z., Chang M., Qi H., Lim J., Yang M., Lyu S. (2015). DETRAC: A new benchmark and protocol for multi-object tracking. arXiv.

[51] Yu F., Chen H., Wang X., Xian W., Chen Y., Liu F., Madhavan V., Darrell T. Bdd100k: A diverse driving dataset for heterogeneous multitask learning. Proceedings of the IEEE/CVF Conference on Computer Vision and Pattern Recognition.

[52] Highway Traffic Videos Dataset. https://www.kaggle.com/datasets/aryashah2k/highway-traffic-videos-dataset.

[53] Traffic-Surveillance-Dataset. https://github.com/gustavovelascoh/traffic-surveillance-dataset.

[54] Tang Z., Naphade M., Liu M.Y., Yang X., Birchfield S., Wang S., Kumar R., Anastasiu D., Hwang J.N. CityFlow: A city-scale benchmark for multi-target multi-camera vehicle tracking and re-identification. Proceedings of the IEEE/CVF Conference on Computer Vision and Pattern Recognition.

[55] DZ Computer Vision (2021). Traffic Count, Monitoring with Computer Vision. 4K, UHD, HD. https://www.youtube.com/watch?v=2kYpqSMqrzg.

[56] Sboukraa I. (2023). Car Object Detection in Road Traffic. https://www.kaggle.com/datasets/boukraailyesali/traffic-road-object-detection-dataset-using-yolo.

[57] Opencv-Python. https://pypi.org/project/opencv-python/.

[58] Garcia-Garcia B., Bouwmans T., Silva A.J.R. (2020). Background subtraction in real applications: Challenges, current models and future directions. Comput. Sci. Rev..

[59] Siami-Namini S., Tavakoli N., Namin A.S. A comparison of ARIMA and LSTM in forecasting time series. Proceedings of the 2018 17th IEEE International Conference on Machine Learning and Applications (ICMLA).

[60] Cascante-Bonilla P., Shehada K., Smith J.S., Doveh S., Kim D., Panda R., Varol G., Oliva A., Ordonez V., Feris R. Going Beyond Nouns with Vision & Language Models Using Synthetic Data. Proceedings of the IEEE/CVF International Conference on Computer Vision (ICCV).

[61] Rekavandi A.M., Rashidi S., Boussaid F., Hoefs S., Akbas E., bennamoun M. (2023). Transformers in Small Object Detection: A Benchmark and Survey of State-of-the-Art. arXiv.

[62] Rekavandi A.M., Xu L., Boussaid F., Seghouane A.K., Hoefs S., Bennamoun M. (2022). A Guide to Image and Video based Small Object Detection using Deep Learning: Case Study of Maritime Surveillance. arXiv.

[63] Coluccia A., Fascista A., Schumann A., Sommer L., Dimou A., Zarpalas D., Akyon F.C., Eryuksel O., Ozfuttu K.A., Altinuc S.O. Drone-vs-bird detection challenge at IEEE AVSS2021. Proceedings of the IEEE International Conference on Advanced Video and Signal Based Surveillance (AVSS).

